
JOURNAL INFORMATION
==============================
NLM Title Abbreviation: Med Chir Rev
NLM Journal ID: 0034617

The Medico-Chirurgical Review

Publisher: Biomedical Journal Digitization Project

ARTICLE INFORMATION
==============================
PMCID: PMC4966178
PMID: 29917601
Article version: 1
Subjects: Articles

Hospital Practice

Print publication date: 1828 Jul 1
Volume: 9
Issue: 17
First page: 241
Last page: 264
1828 Jun 7
License: This work is free of known copyright restrictions. For more information, please see PMC Back Issue Digitization.
License URL: https://creativecommons.org/publicdomain/mark/1.0/

==============================

### HOSPITAL PRACTICE.

86. Cases intended to illustrate the Application and Utility of the Stethoscope. By Richard Townsend, M.D. Medical Inspector of the House of Industry. [Dublin Transactions, Vol. V.]

The object of Dr. Townsend's report is to prove that "auscultation is capable of affording an extraordinary degree of precision in recognizing and distinguishing the most complicated forms of pulmonary disease—and that such information necessarily leads to practically beneficial results." Dr. Townsend does not conceal his mistakes; but candidly attributes them to carelessness or incorrectness on the part of the auscultator, rather than to faulty principle in auscultation itself. Independently of the stethoscope, however, the cases here detailed are pathologically interesting, and being authentic documents from public institutions, are the more valuable on that account.

*Case* 1. On the 25th March, Dr. T. visited, at the request of Dr. Cheyne, a tall dragoon, aged 30, then in the Royal Infirmary. His complaint had commenced in the preceding October, with cough, pain in the chest, and diarrhœa; for which he was bled, blistered, &c. The disease frequently recurred, and was as frequently treated in the same manner. When visited, he was walking about the room, but was easily put out of breath, and was emaciated. Had profuse night-sweats; diarrhœa; thirst; pulse 120, small and vibrating—respirations 30 in the minute—sputa apparently mucous, and reported to have diminished considerably within the last three weeks, during which period the dyspnœa had increased.

"On viewing the thorax, the right side appeared considerably more dilated than the left, especially anteriorly and laterally, at its lower half. Percussion employed over the dilated surface elicited a clear hollow sound. In this space too the respiratory murmur was perfectly inaudible; but immediately after coughing, a peculiar sound, resembling the vibrations of a porcelain jar, when gently struck, (*tintement metallique*,) was distinctly heard in a space corresponding to the posterior convexities of the sixth, seventh, and eighth ribs. This sound was not produced either by inspiration or speaking.

"Succussion did not produce the sound of fluctuation, although the patient said he felt water dashing against his side. In the superior part of the same side of the chest (the right) the dilatation was scarcely, if at all perceptible. The sound, on percussion, not particularly sonorous, and the respiratory murmur audible posteriorly.

"At the left side the sound on percussion was natural, though considerably duller than at the right. Respiration was distinctly audible all over the lung's surface, except in the space corresponding to the superior lobe, where cavernous respiration and cough, with perfect pectoriloquy, were heard distinctly."

Diagnosis. A tubercular cavity in the upper lobe of the left lung. The dilatation of the right side of the chest is produced by pneumo-thorax—the air in the cavity of the pleura proceeds from a communication between the bronchia and that cavity, as proved by the co-existence of the tintement metallique. The medium of communication is conceived to be a tubercular cavity. The detailed diagnosis, of which the above is the substance, was written down, and given to Dr. Cheyne, on the evening of our author's first visit.

On the 1st of April we find the report as follows:—

"Pain of the left side removed by the application of a blister; dyspnœa increased; number of respirations 38; diarrhœa; profuse night-sweats.

"At the right side, sound on percussion is become dull posteriorly in the space corresponding to the inferior portion of the thoracic cavity, where no sound whatever can be heard. *Fluid accumulates.* Above the eighth rib the sound on percussion is hollow, the ordinary inspirations sound like blowing into a bottle. Expiration is followed by a musical sound, resembling the vibrations of a fine wire chord. Coughing produces a peculiar sound, exactly similar to the ringing of a porcelain jar; the voice much more obscurely so.

"On making the patient sit up in bed, and shaking him gently by the shoulder, (the Stethoscope being applied posteriorly about the convexity of the seventh rib,)

a fluid is clearly heard dashing against the sides of the thorax.

"The patient is sensible of this fluctuation, says *he* hears it, though *I* could not without applying my ear. When he suddenly rises from the recumbent posture, three or four drops are heard to fall successively from above on the surface of a fluid. This sound also is most distinctly heard over the seventh rib posteriorly.

"In the left lung, pectoriloquy, &c. as before.

"Feels no pain whatever in right side. I, in consequence, added to my former diagnosis, that the constant absence of pain probably proceeded from a thick coating of albuminous exudation, the product of chronic inflammation, which lines the pleura, and defends it from the contact of the air."

*5th April.* The thorax is more distended—all the intercostal spaces are protruded—signs furnished by auscultation and percussion the same as before, and they were verified by Drs. Brown and Graves, who carefully examined the patient.

*11th April.* Emaciation and debility progressive—night-sweats—stethoscopic sounds as before, except that they are now heard anteriorly, as well as posteriorly, and as high as the third rib. Below the 7th rib, the sound on percussion, is dull, and the respiration extinct.

"I think it right to mention, that though I employed the Stethoscope to ascertain the precise relative situation of the air and fluid, yet all the sounds above recorded were incomparably more evident to the naked ear."*

Death terminated the scene on the 14th April, and the following are the minutes of dissection, made in the presence of Drs. Cheyne and Stack.

"*Thorax.* The right side. A trocar was introduced between the fifth and sixth ribs near their junction with their cartilages; an immediate rush of air followed, part of which I succeeded in collecting in a large phial filled with water, and inverted over the canula.

On removing the sternum, a vast unoccupied space was observed in the anterior part of the thorax, capable of containing fully two quarts of water. This space had been occupied by air, which may consequently be estimated at that quantity. The lung just appeared above the surface of the fluid, which occupied the posterior region of the thorax; it was closely compressed against the spine, and seemed reduced to one-third of its natural dimensions. The fluid effused might be in quantity about two quarts, was of a yellowish green colour, tolerably clear at its surface, but rendered turbid at bottom by numerous fragments of opaque, puriform flocculi of albumen.

"Before touching the lung, in order to guard against an accidental formation of the opening, which I expected to find, an incision was made into the trachea, and the pipe of a pair of bellows introduced. The air passed freely through the lung, and appeared in bubbles at the surface of the fluid, in which it was immersed.

"The fluid being removed, the upper lobe of the lung was found in close contact with, and firmly attached to the costal pleura.

"The whole surface of the lung, except where it was attached, was coated with an albuminous exudation of a dirty white colour, of several lines thickness, its surface wrinkled, not unlike the rind of a shrivelled apple. The costal, mediastinal, and diaphragmatic pleuræ, were still more thickly coated with this exudation, which, though firmly attached to the subjacent pleura, and apparently incorporated with it, might, by careful dissection, be separated from it, leaving the membrane underneath in a state of perfect integrity.

"The lung was now detached; on its anterior surface, about two inches from the summit of the upper lobe, was discovered a fistulous orifice, capable of receiving my little finger, its margin well defined, rounded, and nearly cartilaginous. A probe introduced passed readily

---

* We have been in the habit of almost daily employing the naked ear and the stethoscope, for seven or eight years past, and of estimating them comparatively. The results of our experience are in perfect consonance with the observation recorded in the above extract. Several of our friends, however, give a decided preference to the stethoscope; and although they can give very ingenious acoustic reasons for their preference, yet we cannot allow argument to predominate over the evidence of our own senses.—*Rev.*

through a series of small tubercular cavities into one of the principal bronchia. At intervals of half an inch below this fistulous orifice existed three small oval superficial ulcers, which, on close examination, did not appear to communicate with the bronchia. They were evidently formed by softened tubercles, developed immediately under the pleura; for on different parts of the lung's surface there were several similar oval nests of tubercles, some not yet softened, others quite soft, and elevating the pleura, through which they had not as yet formed a passage.

"Posteriorly near the root of the lung, and about the base of the superior lobe, immediately underneath its adhesion to the pleura, was another fistulous opening, of half an inch in diameter, which communicated by a long sinuous passage with a large tubercular abscess occupying nearly the whole upper lobe. This passage was lined all through by a highly vascular membrane, exactly similar to that which lined the tubercular abscess, having its surface coated with a layer of lymph. Into this vast abscess was also traced one of the principal bronchial divisions; its entry into the cavity was within a few lines of that of the sinuous passage above described.

"The middle and lower lobes contained several tubercles. The bronchial glands also were much enlarged, and studded with tubercles.

"The left side of chest. This lung was studded throughout with granular tubercles of the size of duck-shot. In the superior lobe was found one cavity, capable of containing a large filbert, and communicating with two or three smaller ones. In the middle lobe the tubercles were all opaque and whitish. In the inferior many of them were in the first, or greyish, semi-transparent stage.

"The heart was small, flaccid, and pale."

The inferior margin of the liver descended to the crest of the ilium—mucous membrane of the stomach reduced to a pulpy mass, which was easily scraped off with a scalpel. There was ulceration of the ileum near the cæcum.

"This case of pleurisy with pneumothorax, differs from any other that I have seen recorded, in the total absence of pain in the side affected, in not having its commencement marked by any violent or sudden symptoms of dyspnœa or pain, and also in this, that the patient was able almost to the time of his death to dress himself and sit up; whereas M. Laennec expressly states, that in all the cases which he had seen, the patients were excessively oppressed, and unable to quit their beds. Indeed he lays down these circumstances, as adjuvant diagnostic marks, whereby to distinguish this disease from emphysema. But in this instance such minor distinguishing features were not necessary. The great distention of the right side of chest, its remarkable sonoriety, the total absence of respiration, unless where the lung was attached, the extraordinary development of the pathognomonic signs, all established, beyond the possibility of doubt, the precise nature of the disease. I shall only add, that the dull sound supervening inferiorly, and gradually ascending, the increasing capacity and sonoriety of the superior part of the thorax, together with the increased extent of surface over which the pathognomonic sounds were heard, did all, and each of them, mark exactly the progress of the disease from day to day, up to the period of its fatal termination."

The lung, preserved in spirits, was presented to the Association, when the paper was read.

We shall take occasional opportunities of presenting to our readers several other interesting cases, recorded in this communication, as they are all insulated cases, and not necessarily connected by any inseparable links. But we cannot help adverting, in this instance, to the contrast between the Dublin and London practitioners, as presented in the reports from public institutions. On this side of the Channel, (with few exceptions,) Plutus, and not Apollo, is the god of idolatry, before which the physician and surgeon prostrate themselves, morning, noon, and night! Speaking *generally*, every one of those muscles which contribute to the expression of an exquisite *sneer*, is thrown into rigid contraction, the moment that any pathological enquiry, beyond money-making routinism, is broached! *Post-mortem* investigations are shunned, as taking up that time which is more profitably employed in manœuvring and drugging the public—

*auscultation* is voted to be not merely a bubble, but a *bore*, that requires study, in a science where no other study *ought* to be introduced or cherished, but that which enables the doctor to make the most money in the shortest space of time—every improvement in diagnosis is assailed, even through the medium of the PRESS, by every species of obloquy and ridicule—while ROUTINE EMPIRICISM becomes the order of the day! Look at the cases published in our journals by private practitioners—and the reports from hospitals. Almost all are trying to cure diseases which they *do not know*—while very few indeed are trying to know those diseases which *cannot be cured*. It will be said that, "it is of little use to have a very accurate knowledge of those changes of structure that are beyond the reach of art." Nothing is more false, nothing more injurious than this sentiment. It is the duty of medical men to *mitigate* sufferings where they cannot remove the cause—to *prolong* life, where they cannot ultimately save it. These duties cannot be performed without an accurate knowledge of pathology—that is, without knowing the deviation from sound structure, *while the patient is living*. For want of this knowledge, thousands are daily injured and their lives curtailed by active measures and the exhibition of potent drugs, under the idea that diseases are functional or curable, when they are organic and incurable—consequently where nothing but the mildest means should be used for their mitigation. This we know is unpalatable doctrine for the routine practitioners; but the "march of intellect," beyond the pale of medicine, is enabling the public to look over the walls of colleges, hospitals, and all those places formerly sacred to the *art* and *mystery* of physic. We are greatly deceived if medical men be not forced, not merely to keep pace with the progress of knowledge generally, but to keep their own science in *advance*. Nothing but the most unwearied exertions, the most unconquerable zeal can enable them to effect this object. Self-love, and some other passions, which shall be nameless, will raise an outcry against these sentiments, as illiberal, and as depreciating our dear country. But no clamour of this kind shall deter us from doing what we conceive to be our duty.

87. HOMICIDAL AND INFANTICIDAL MONOMANIA.

The following melancholy and remarkable cases are recorded by Professor Outrepont of Wurtzburg, in a late German Journal.

*Case* 1. The individual was a surgeon who had served in several campaigns against the French, and who afterwards studied midwifery under the aforesaid Professor. He always appeared of a lively and cheerful disposition, till certain pecuniary matters ruffled his temper and rendered him thoughtful and melancholy. He was now frequently observed to be studying the Scriptures, and reciting passages from the Bible. He was happily married, and had four children. One morning he summoned his wife and children into the court-yard of his house, and there informed them that it was his intention to destroy them all, and afterwards himself! He descanted coolly on the propriety of homicide, and told his wife she must first be a spectatress of the destruction of her children, and then her own turn would come. The woman appears to have been possessed of great presence of mind, and acted with great prudence on such a trying occasion. She entirely coincided in the justness of her husband's sentiments, and cheerfully agreed to the proposed tragedy. But she appeared suddenly to recollect that it would be proper for herself as well as her children, to confess and take the sacrament, previously to appearing before their final judge—a ceremony which would necessarily require several days' preparation. The monomaniac replied that this was a reasonable and proper procedure; but, in the mean time, it would be absolutely necessary that he took some person's life that day. For the purpose of effecting this, he instantly set off for Salzbourg. His wife, having placed the children in security, made the best of her way to the above-mentioned town, and went directly to Professor O. the friend of her husband, for advice. The monomaniac had already been there, and not finding the Professor at home, had gone away. The woman now recollected, and told the Professor, that her husband had threatened *his* life, for some imaginary slight which he had experienced; but, at that time, she thought he was in jest. About mid-day, the monomaniac came back to the Professor's residence, and appeared quite calm and peaceable. The Professor invited him to go and see the hospital of the town, where he had a curious dissection to make, and they sat down to eat some refreshments before proceeding thither. At this repast, the monomaniac informed his host that he had lately been most immoderately disposed to commit homicide—and that he had actually murdered a peasant that morning on his way to the town! He confessed also that he had entered a coffee-house for the purpose of committing a second act of this kind, but had been diverted from his purpose. The murder of the peasant was a fiction, as was afterwards proved. The Professor now turned the discourse to other subjects—and on all other topics, the monomaniac was perfectly rational. They now set off for the hospital, and in their way thither, the monomaniac met with an old acquaintance and fellow-campaigner. While they were greeting each other, the monomaniac suddenly struck his friend a violent blow on the pit of the stomach, exclaiming, in a burst of laughter, that he had done for him, as he had hit the cœliac plexus! The Professor reprimanded him in strong terms for this dishonourable and cruel act, at which the monomaniac was much surprised—and informed his preceptor that he was irresistibly led to commit homicide, and cared not who was the victim of this propensity. The Professor now asked him somewhat tauntingly, if he had not a design against *his* life? The monomaniac acknowledged it; but added that he had sufficient control over himself to prevent the destruction of his benefactor. The Professor took his arm, and they proceeded to the hospital, where the monomaniac was immediately confined. He almost instantly became furiously maniacal, and, in a few months afterwards died.

Another case is related by the same author, which merits the attention of the medico-legal inquirer.

*Case* 2. A female who was received into the Lying-in-hospital, to which our author was chief accoucheur, requested a private conference with the doctor previously to her accouchement. She appeared in a state of great agitation and embarrassment, and presented many of those phenomena which usher in the *mania post partum.* When the attendants were all withdrawn, she begged earnestly that Dr. O. would not leave her in the same chamber with other women and their *infants*, as it would be utterly impossible for her to resist the propensity she felt to destroy the *latter*. Her request was granted, and she was very attentively watched. Her delivery was easy, and the child was kept from her, and afterwards sent to her mother. The young woman, on leaving the hospital, went as a servant in the town, and would not return to her mother's house, lest she should be tempted to destroy her infant. She declared that the sight of a very young infant kindled up an irresistible propensity to destroy its life. This young woman was a peasant, who had been seduced, and who had never led a dissolute life, or was, in any way, of corrupt manners. She had not received any reproaches or ill treatment from her parents, during pregnancy—nor was there the least cause for anxiety on account of the child, as her mother had engaged to provide for it. In fact, our author could discover no tangible cause for this infanticidal disposition. She entered into the service of a clergyman, and enjoyed good health. Some time afterwards our author questioned her on the above subject, and she informed him that she had nearly lost all propensity to infanticide. We have no doubt that it is in this way many cases of infanticide occur, where verdicts are given against the mother by an ignorant and prejudiced jury.

---

88. CLINICAL REPORT ON FEVER. By Dr. Jackson, Physician to the Philadelphia City and County Almshouse.

We are rejoiced to see that our transatlantic brethren are eagerly encouraging the publication of hospital-reports. The plan which we have so often recommended, (as pursued in Paris) of delegating the task of reporting to some able and competent pupil, is pretty generally adopted, as far as we can learn, in the United States; and we wish it every success. We select the present report, published in our highly respected contemporary, (the American Journal of the Medical Sciences) for a short article, as it exemplifies the progress of the doctrine of Broussais beyond the Atlantic. The fever here reported on, occurred in the months of August, September, and and October of 1827. The patients were chiefly newly arrived emigrants from Ireland, "who were generally persons of temperate habits and good constitutions"—and the character of the disease was, no doubt, considerably modified, if not produced, by the changes in diet and modes of life of the sufferers. From food, consisting chiefly of potatoes, oatmeal, and butter-milk, the emigrants were unavoidably put on salted meat, during the voyage, and they indulged in substantial animal food meals after their arrival in America. The autumnal season also contributed to the production of the disease. "This fever," says Dr. Jackson, "was a gastro-enteritis, with meningeal or other complications." The cases detailed from the hospital-books, (and they are very numerous) certainly shew that the mucous membrane of the stomach and bowels was the chief seat of the disease, and the treatment consisted, almost uniformly, in local depletion by leeches, starvation, diluents, and antimonial diaphoretics. When the inflammatory irritation was propagated to the brain or its meninges, the local depletion was carried to the head or neck, and so on, according to circumstances. The following extract contains many sensible observations, and will enable our readers to form a very good idea of Dr. Jackson's views and practice. We recommend it to the especial attention of those who are deeply imbued with the Hamiltonian and Abernethian doctrines in this country—admitting, at the same time, that the practice of drastic purgation is much less detrimental in northern than in hot climates, because the head is more frequently the seat of inflammatory action in the former than in the latter countries.

"The preceding cases recorded by R. L. FEARN, student, are examples of common remittent fever or gastro-enteritis, so common in the commencement of warm weather. They manifested cerebral symptoms, the invariable concomitants of acute gastric inflammation. The cerebral sympathetic irritation did not acquire an intensity sufficient to become independent of the gastric inflammation in either case. In Mary, on the first of August, the cerebral symptoms rapidly increased, and the irritation of the arachnoid and pia mater threatened to take the lead in the train of morbid phenomena. The application of leeches along the jugulars, and around the base of the head gave an immediate check to this complication.

"The prescription directed in both cases previous to my visit to the patients, though a common formula in fevers was discontinued, that the existing inflammation of the mucous tissue of the stomach and intestines might be attacked by the most direct and appropriate means. That formula is certainly far preferable to the active and drastic cathartics frequently resorted to, and the employment of which has been so much misapplied and abused since the publication of Hamilton's work on Purgatives. Still its immediate direct mode of operation is that of an irritant to the surface to which it is applied. This irritation it is true, under common circumstances generally produces secretion from the exhalent vessels and mucous follicles of the mucous tissue of the intestines, and a purgative operation is the consequence. This irritation and secretion are the essential element in the action of a purgative. But it is a well demonstrated fact, that an irritant applied to an irritated surface, will as frequently dry up and prevent secretion as excite it.

"Purgatives in the acute stage of fevers attended with inflammation of the gastro-intestinal mucous tissue, when they do excite copious secretions, counteract their own injurious tendency, and often arrest or diminish the inflammation of this membrane, when it is not deeply radicated or extensively diffused. It is, however, in the experience of every practitioner, that active purgatives in fevers frequently fail to produce evacuations: they cannot bring forth secretions from a highly inflamed surface, whose actions transcend the point at which secretion can take place. The irritation of the purgative, under such circumstances, is added to that already existing, and all the symptoms are aggravated.

"The excitement of secretions and evacuations by purgatives in fevers very frequently, instead of alleviating the condition of the patient, renders it much worse. It is not uncommon that an apparent amendment will ensue from very free, copious, intestinal secretions procured by purgatives, for twenty or more hours, to which succeeds an aggravation of the precursory symptoms. The depletion operated in this manner on the general circulation, serves temporarily to tranquillize the sympathetic irritation of the central organ of the circulation; and, in consequence, the febrile symptoms—frequence and force of pulse, heat of skin, &c. depending on that condition of the heart, are for a short period abated. The symptoms of the local irritation may for a time be smothered from the extent of the evacuations, but they break forth with increased violence.

"The administration of active purgatives in fevers, often proves injurious, by rendering extensive a morbid irritation which may be very limited. The purgative, irritating zone after zone of the mucous coat of the alimentary canal, as it passes along, becomes a conductor of the inflammation, restricted primarily to a small portion of this structure, and spreads it over nearly its whole surface. In the tegumentary tissue of which the digestive mucous membrane is a part, erythemoid inflammation, as it is called, has a strong disposition to propagate itself, even without a provocative. From this cause, those labouring under fevers, who are treated by drastic cathartics or other irritants, have the abdomen to become tumid, hard, painful to pressure, tympanitic—to be meteorized as these collective symptoms were formerly designated; the bowels are costive, and very little flatus is discharged. Here the inflammation has not passed beyond the ileo-cœcal valve and penetrated into the large intestines. This does occur, however, sometimes, and then is added to the foregoing symptoms, a diarrhœa with varied discharges of different hues and appearances; brown, white, black, and bloody, and involuntary stools, according to the intensity of the inflammatory action. When purgatives and other irritants of the gastro-intestinal mucous membrane are abstained from, or but sparingly employed, these symptoms are of very rare occurrence, in fevers even of a high grade of action, and which may prove fatal by the disorganization of the mucous membrane of the stomach, effusion on the brain, &c.

"From these principles I have adopted the practice, the oldest in medicine, and if authority be entitled to consideration, sustained by the weightiest in our profession, of abstaining generally from purgatives in the commencement of acute fevers, and of combating, by directly debilitating remedies, the excessive actions in the tissues in which they are located. From a comparative experience of several years practice on both systems, I am daily more convinced of the superiority of that I now pursue. Those cases of fever, in which the capillary irritation is of a light grade, or a limited extent, and in which purgatives prove successful remedies, the unmixed, positive antiphlogistic treatment, established on anatomical and physiological principles, will be found in the great majority to be more speedily cured, without the slightest risk being incurred of converting them into more formidable affections."

We are convinced that there is much good sense and solid practical observation in the foregoing remarks. The report embraces a great variety of complications in this fever, but the principles above stated were uniformly acted on, and the success appears to have been as great as could possibly be expected under existing circumstances.

---

### 89. M. Lassere on Cancer of the Face.

Of all the ills "which flesh is heir to," none, perhaps, are of such a melancholy nature as cancer; a disease most racking to endure, afflicting, and even disgusting to witness, and too often setting at defiance the best directed efforts of medical or surgical skill. M. Lassere, the author of the case we are about to notice, congratulates himself on the success which has attended the *médecine physiologique* in this unhappy malady, but we fear that on the Continent, a greater latitude is given to the term, schirrhus, than in this country, and a too easy credence lent to instances of cure.

*Case.* Adrien Chabaunes, æt. 19, in the month of March, 1822, had a molar tooth extracted. A good deal of difficulty was experienced during the operation, and the alveolar process and gum were much injured. Inflammation of the mucous membrane of the mouth followed, and was relieved by astringents, but the gums remained swollen, and bled frequently. Small pustules also formed, especially on the upper lip; and, towards the end of May, one of these became very painful, and degenerated into an ulcer. A young physician who was consulted, prescribed soothing applications, but the ulceration spread, attended with excruciating pains, and in the month of August, our author was called in. At this time, the ulcer extended from the margin of the upper lip to the septum nasi, and nearly half an inch on either side; the surface being of a dirty red colour, the edges hard, irregular, everted, and exceedingly painful to the touch; the lip swoln, the surrounding parts inflamed. The discharge consisted of an offensive mixture of blood and pus, and hæmorrhage took place at each dressing from the edges of the wound. Within the ten days previous to M. Lassere's visit, he was informed that the disease had increased upwards of a third; the *morale* was affected, the pulse small and frequent.

Our author considered the case as one of cancer, and drew a gloomy prognosis in consequence; however, he ordered eight leeches to be applied on each side of the ulcer, and repeated every other day—a carrot poultice made with decoction of hen-bane and garden night-shade, to be renewed thrice daily—a warm bath during the day, and a pill morning and evening, composed of one grain of extract of hemlock, and a quarter of a grain of extract of black hellebore. This, with a vegetable diet, and an occasional enema composed the treatment, which was persisted in for six weeks, at the end of which time the wound had assumed a healthy appearance, and had even begun to cicatrize. The vegetable was now changed for an animal diet, with a moderate quantity of wine, the narcotic pills discontinued, and those of "Belloste" substituted for them, and finally, the leeches were applied less often, and fewer in number. In seven days more the poultice was left off, and the wound dressed, and in a fortnight from this period, the ulcer had entirely healed. The lip, however, still remained thicker than it should be, which annoyed the patient and induced him to be constantly pulling it about and pressing it between his fingers. The consequences were soon obvious—the lip swelled again and became inflamed—the ulcer broke out afresh, and this time proceeded with such rapidity, that in a few days it had acquired its former volume. Three months elapsed before our author saw the patient, and at that time "the ravages of the disease were frightful." It had extended upwards on the left side, and almost destroyed the ala nasi, then descended along the ramus of the lower jaw to the chin, whence it passed upwards on the right to the right side of the nose, which, however, was as yet unhurt. The same plan of treatment which had been so successful in the first instance was resumed, and soon stopped the further progress of the disease, but full two months and a half elapsed before cicatrization had commenced in the ulcer. The Belloste pills, which appear to be a mercurial preparation, were then had recourse to, but it was necessary to increase the dose so high as to induce fears of salivation taking place. Under these circumstances, there being much anemia, and the mucous membrane of the mouth blanched and discoloured, M. Lassere determined on the administration of the tincture of iodine, beginning with six drops three times a day, and gradually increasing the dose. A seton at the same time was inserted in the nucha. In six weeks the ulcer was entirely healed, but for the space of three months more the patient wore a bandage on the part, to protect it from the atmospheric influence, and external injury.

This is an interesting case, but we cannot say that we believe it to have been one of cancer. That it was a foul, unhealthy ulcer, which, under improper treatment, or no treatment at all, would have gone on to mischief, and terminated in malignant disease, we think is highly probable, but still we do not think the characters were by any means those of scirrhus. The case serves well to show the utility of local depletion and soothing treatment, in these unhealthy ulcerations about the gums, which occasionally accompany carious teeth, or follow injury to the alveolar process in their extraction.

---

### 90. Open Foramen Ovale in the Adult.

This case, which is published by Dr. John Crampton, in the recent volume of Dublin Transactions, is prefaced by some remarks on the "facilities afforded by the Dublin Hospitals for investigating the nature of diseases after death." He assures us, and indeed we know it to be true, that—"the public, and even the lower orders seem every day to lose those prejudices which were formerly entertained against anatomical examinations." It is curious that the "wild Irish," as John Bull calls his Western neighbours, should get the start of their masters, the English, in emancipation from the shackles of prejudice! When, indeed, we hear a grave and learned judge, descanting on the enormous crime of dissecting a human body, knowing it to have been disinterred (and how is it to be otherwise obtained?) we cannot wonder at the inveteracy of those prejudices which disgrace the highest as well as the lowest orders of society in this country. But we hope for better times.

A young man, aged 18 years, was admitted into the Hardwicke Hospital, on the 6th Feb. 1827, as a fever patient, shewing thoracic inflammation, with cough, difficult breathing, and bloody expectoration. By depletion he was rendered convalescent by the 14th, but, on premature exposure to cold, he was seized with acute rheumatism, which became ultimately translated to the chest, especially to the heart, where great distress was felt. The more acute symptoms were allayed by active depletion; but on the 27th Feb. we find the patient with pain in the region, severe cough with croupy sound—copious muco-purulent expectoration—great difficulty of breathing. He now stated that he had been subject, for some years, to pain in the left side, especially after running, or any active exertion. The pulse was rapid and irregular—countenance pale—feet œdematous. "The stethoscope indicated acute bronchitis, with hypertrophy, and disordered action of the heart, the motions of that organ being tumultuous and irregular." He lingered till the 10th March, when he expired.

*Dissection.* "The thoracic cavity was examined on the 12th. On opening it the lungs did not collapse, but the external appearance of this organ was healthy; the parenchymatous tissue exhibited some portions red, congested, and evidently inflamed; the bronchia vascular, and filled with a muco-purulent effusion.

"Pericardium contained about two ounces and a half of fluid, in which a considerable quantity of coagulable lymph was seen; inner surface of this membrane not inflamed, but that portion of it which is reflected over the heart showed marks of inflammation in several places; both ventricles were much enlarged, exhibiting considerable hypertrophy; the semilunar aortic valves showed recent fleshy or *cauliflower* excrescences attached to all of them. Both the ventricles were quite filled with a dense, white coagulum, firmly attached to their parietes. The commencement of the aorta appeared unusually narrow, in proportion to the heart and to the subject; that of the pulmonary artery greatly enlarged. The auricles of the heart were also in a state of considerable dilatation: but the circumstance which attracted attention most, was, that the foramen ovale was open; but the manner of this opening must be more fully described. The septum between the auricles exhibited an oval depression, or attenuated space of about one-third of an inch in diameter, guarded only by a thin membrane; but at one side it was evidently pervious and open, with a rounded and thickened edge. This membrane acted like a curtain or valve; when viewed or pressed from the left auricle it was closed, the curtain or membrane pressing close, and overlapping the opening; viewed from the right auricular cavity, or touched with a probe, it opened, and allowed a free passage, fully as large as a goose's quill, compressed so as to exhibit an elongated or oval section. The blow-pipe exhibited the same difference of a closed or open space, as it was used from the left or right side of the auricular septum."

Dr. Crampton enters into a long train of reflections on the consequences which must have resulted from this valvular opening between the two auricles of the heart, including the "dark unrenovated blood"—the "tumult and distress felt in the heart and respiratory organs, &c." But we see no evidence of this unrenovated blood, before the last fatal illness—nor even then; and, as to the tumult and distress about the heart, we attribute *them* to the inflammation resulting from metastasis of acute rheumatism. Dr. Crampton tells us that, in such a case as the above, there is only a part of the venous blood sent through the lungs for renovation, a portion going through the preternatural opening into the left auricle, "from whence it is prevented from returning by pressing back against, and closing the valvular curtain already described." We are much surprised at this reasoning. Does Dr. Crampton remember, that the two auricles act *at the same instant*, and not in succession? If the right auricle, indeed, acted while the *left* was passive, some blood might go through the valvular opening into the *latter*—and if the left acted subsequently, while the right was passive, the valve might still prevent the return. But there is no such thing. The two auricles act at the same instant, and both sides of the septum auriculorum are equally pressed, so that, even when there is a wide and patent opening, there is very little intermixture of blood, where the heart otherwise preserves its relative proportions and capacities. It is where the strength of the one auricle predominates over that of the other, or where any obstruction to the natural issue of blood from an auricle takes place, that the foramen ovale produces the admixture in question. Thus, supposing there was a contraction of the opening between the left auricle and ventricle, such obstruction might cause some blood to pass through the foramen ovale, if it remained open. But in a natural and healthy condition of the chambers, we maintain, that such opening would produce little inconvenience, under ordinary states of the circulation. Such appears to have been the case in the above instance. There was no symptom of blue disease—at least, Dr. Crampton has recorded none. This explanation accounts for the occurrence of open foramen ovale, in people who had evinced no appearance of blue disease during life. In a postscript to this paper, Dr. C. appears to recognize the principle which we have stated—for, after mentioning a patient, aged 34, who died in the hospital, and whose foramen ovale was found open, he says, the valve, as in the other case, overlapped and protected the aperture, so that no blood was likely to pass, *unless the contracting power of one side of the heart prevailed over that of the other*. This is the view which we gave of the malformation in question, many years ago. The same reasoning applies to unnatural openings in the septum ventriculorum.

---

91. HOSPITAL REPORT OF M. CHOMEL, FROM LA CHARITÉ, BETWEEN THE 1ST OF FEBRUARY AND THE 1ST OF SEPTEMBER, 1827. Reported by M. DE LAGARDE.—REV. MED.

1. PERIPNEUMONY. The total number of cases [of all kinds] treated in the above period are 281, of whom 39 died. The proportion of acute diseases was greater than usual, as compared with the chronic. To shew how uncertain is the ratio of mortality in the same disease at different periods, we may remark, that out of 18 cases of peripneumony, occurring in the spring quarter of the above period, six died—while in an equal number of peripneumonies occurring in the Summer, there was not a single death. The treatment adopted by M. Chomel (who is an excellent practitioner) was active depletion, adapted to the violence of the symptoms and strength of the patient. But although this treatment was put in force, even from the earliest dawn of the inflammation, the latter could not always be checked; but pursued its course to a fatal termination. Large doses of antimony were tried in some cases, with success—in others, without good effects.

2. INTERMITTENTS. Twenty-four patients labouring under intermittent fever were admitted during the half-year. The types were various, and some were complicated with slight inflammations, not requiring any active treatment. A few days were always allowed to pass before the febrifuge was administered. In eleven cases, the cure was spontaneous, without any medicine, and merely from change of scene and proper diet. When the ague resisted this process, the quinine was given in doses of not less than six grains in the 24 hours, with complete success.

3. ANEURISM. One case of aneurism of the arch of the aorta, with pulsating tumour below the left clavicle presented itself. The patient was a washerwoman, aged 38 years. The plan of Valsalva was tried, but without success, and the patient left the hospital, tired out with the starvation and depletion which she underwent there, without any apparent benefit.

4. GASTRIC COMPLAINTS. Several patients labouring under derangements of the stomach [embarras gastrique] were treated during the semestre. Many were cured by low diet, repose, and acidulated drink. In some cases it was necessary to prescribe emetics. In a certain proportion of cases, these were extremely serviceable, and removed the symptoms very quickly—in others, the emetics were injurious—" a fact which proves that stomach affections are not all of the same nature, and to be cured by a routine treatment."

5. ACUTE RHEUMATISM. Most of these cases were managed by gentle antiphlogistics and low diet, requiring from two to four weeks for cure. In one case, which was very severe, and where almost all the joints were swelled and inflamed, the large doses of tartar-emetic were employed (six grains a day) without *much*, indeed with scarcely any good effect. The TOLERANCE (a term applied to the period when the antimony ceases to cause sickness) was established on the second day; but little or no progress was made, the rheumatic inflammation rather shifting its seat than quitting its hold. After a month's sojourn in the hospital, the patient went away, by no means cured. It does not appear that our Gallic neighbours venture much on bark, arsenic, mercury, or other heroic remedy in this distressing disease.

6. AMENORRHŒA. The general practice in France is to apply fifteen or twenty leeches to the pudendum. M. Chomel prefers applying three or four daily, for five or six days, at the expected menstrual periods, in order to imitate more nearly the process of nature. Previously to the expected epoch, he orders cupping-glasses (dry) to the upper and inner part of the thighs, and warm vapour baths to the lower part of the body.

We shall now proceed to analyse some of the cases detailed in this report.

7. PNEUMONIA CARDITIS. A plumber, aged 40 years, previously strong and healthy, with the exception of some catarrhal affections, felt a shiver, on the evening of the 6th February, 1827, followed by pain in his right side. He went to work the next day, but on the 3d he was obliged to take to his bed. On the 10th Feb. he entered the hospital. Below the right nipple there was acute pain, increased by deep inspiration, with inability to lie on that side. The cough was frequent, the expectoration free and mucous, the sound, on percussion, rather dull, even on the left side. In the right side of the chest the "râle sonore" and the "râle crepitant" were heard in several points. In the posterior portions of lung, in this side, the respiration was pretty natural. In the *left* side the respiration was every where heard. He had been bled the preceding evening, and the blood was highly inflamed. Fifteen leeches to the side affected. Ptisans. 11th. Little alteration. Venesection to ℥xij. was ordered, but not carried into effect. 12th. The symptoms are exasperated, and the sonorous wheeze is heard in the left side of the chest. Bled to twelve ounces. 13th. No diminution of the symptoms—blood not much inflamed. To take six grains of tartar-emetic in the next twenty-fours, in an emulsion. 14th. The patient is more quiet—no alteration in the state of the chest, as indicated by auscultation—four stools from the antimony, but no sickness. Tongue natural—no tenderness at the epigastrium. The antimony to be repeated. 15th. The countenance more sunken. Has had two stools. The antimony to be increased to twelve grains. 16th. Very obscure in the region of the heart, on percussion—respiration very quick—cough and expectoration the same—no stools, no vomiting—pulse 108, and irregular—great debility. Tartar-emetic to be increased to 24 grains. 17th. Had abundant perspiration last night—scarcely any respiration to be heard in the front of the left side. Antimony increased to 36 grains.

We need not pursue the case any farther. The patient died on the 20th of the same month.

*Dissection.* There was no diseased appearance in the head. The left lung was everywhere adherent, and the superior three-fourths of that lung were hepatized—the inferiór portions not quite so, but approaching to that state. The right lung presented the different degrees of hepatization. There was some ulceration in the mucous membrane of the larynx, about the glottis. There was pericarditis, and considerable effusion into the cavity of the pericardium, of almost a purulent fluid. The whole of the heart was covered with false membranes from a line to two lines in thickness. There were several small ulcerations, and other marks of inflammation in the mucous membrane of the œsophagus, stomach, and intestines.

*Reflections.* M. Chomel evidently anticipates the remark that depletion was too sparingly employed at the commencement of this disease. He excuses himself by observing that the two general bleedings produced no mitigation of the symptoms. But what are two bleedings of twelve ounces each? Double the quantity would have been a great deal too little. Besides, the day that was allowed to elapse between the first and second bleeding, was a fatal neglect. Bis dat qui dat cito. But M. Chomel, or his reporter, tells us that, in the same ward, a few days previously, a man had been attacked with pneumonia, and was very vigorously depleted from the beginning, yet still the inflammation went on to a fatal issue. Hence he concludes that there was something specific or malignant in the peripneumonies of the Spring of 1827. There might be so; but we question whether either of these cases presented any just grounds for not carrying depletion to a much greater extent, without attending to any thing but the disorganizing inflammation that was evidently advancing daily on a vital organ.

Whether the large quantities of tartarized antimony which were swallowed during the illness, without producing either vomiting or purging, had any thing to do with the ulceration and inflammation observed in the primæ viæ, we will not pretend to say, since much greater devastations are seen where no medicine of this kind has been administered. But, considering the effects which this substance produces on the external surface, we should not wonder if a corresponding degree of irritation might not be produced by such large exhibitions internally. We shall make room for one more case.

8. MOLLESCENCE OF THE COATS OF THE STOMACH. A married woman, aged 22 years, who had had one child, became troubled with a considerable mænorrhagia in the beginning of the year 1827, at which period she was also exposed to several moral emotions of a distressing nature. Nevertheless she became pregnant, and experienced almost constant malaise—anorexia—thirst—tenderness at the epigastrium after eating—and, finally, vomiting of yellow and bitter matters. It was three days after the commencement of these more serious symptoms that she entered LA CHARITÉ, viz. on the 24th May, 1827. The expression of the countenance was natural, as was the state of the skin and tongue—the pulse was scarcely quickened—thirst very moderate—epigastrium very tender on pressure, but the abdomen soft and indolent. Each day she had ten or twelve vomitings of bilious matters, with some streaks of blood—stools regular. Leeches were applied to the epigastrium, and fomentations, lavements, diluents, &c. were employed, but without success. The vomitings continued—the tenderness of the epigastrium increased—the tongue was sometimes red, or shining—sometimes natural. Opium, for a time, diminished the sickness, but ultimately failed. On the 24th June, the sickness suddenly ceased—and the epigastric pain vanished entirely. But debility and emaciation advanced, and she expired on the 9th July, no vomiting having occurred for a fortnight before dissolution.

DISSECTION. On opening the abdomen, the stomach was found torn from the cardiac orifice to about the middle of its anterior surface; but without any extravasation into the abdomen. A great portion of the mucous membrane of this organ was completely destroyed, and some parts of the muscular and peritoneal coverings were so soft and thin as to be ruptured almost by handling them. There were only a few red patches in the mucous membrane of the intestines. The uterus contained a fœtus of three months.

REMARKS. This was an extremely well-marked case of gastritis, (of the mucous membrane,) as far as pathology was concerned. But it is not a little remarkable that, while such a dreadful disorganization was going forward in a vital viscus, there should be so little febrile disturbance in the system. The pulse and skin scarcely evinced any deviation from a state of health, and the tongue was often natural. The cessation of the vomiting too, for a fortnight before death, was an occurrence not to be expected, according to the ideas which are formed from elementary instruction, and systematic descriptions of diseases. It is from clinical experience, and from faithful clinical reports, that the mind becomes stored with the knowledge of those almost infinite varieties presented in diseases, the want of which knowledge renders the practitioner liable to perpetual error in prognosis and diagnosis. The apparently dry details of a case of this kind are quite wearisome, if not disgusting to the young, and especially to the routine practitioner. But we can tell them, that a careful perusal of such cases is one of the best modes of disciplining the mind for receiving accurate impressions at the bed-side of sickness. There is a very prevalent idea among professional men, that *practice alone* makes the good and successful practitioner. We deny it—and this denial is grounded on more than 30 years of careful observation, not only of diseases, but of men. In all that course of time, we never knew a good and a successful practitioner who did not read and study, as well as observe. It is usual for the lazy man of experience to quote John Hunter, as an example of great eminence, without reading. Not having known John Hunter, we cannot speak as to his *practical* talents; but the foregoing opinion is the result of what we have seen among our own acquaintances, which are not very few. It is fashionable to deride books and study; but, for our own parts, we have no hesitation in affirming, that nine-tenths of our *practical* knowledge would never have been acquired, had it not been for that discipline which results from studying the practical observations of others. This sentiment from grey hairs may probably have some weight with those who think that every thing is to be gained by the *sight* of diseases, and little or nothing from *reflection* excited by reading. Not a day passes—not a day has passed for twenty years, that we have not seen the most outrageous errors committed by men who pride themselves on never consulting any thing but their own *experience*. Such men were born in darkness—live in darkness—and will die in darkness.

---

### 92. TUMOURS WITHIN THE ORBIT.

We resume the review of the cases detailed by Dr. Ballingall, in his Clinical Report, and we must say that we highly approve of the very candid manner in which that Report has been drawn up. The cases bear the impress of fairness and authenticity; are told in a plain, unvarnished style; and are not of that *select* description preferred by *some*, whose object in giving only instances of success, is most pure and disinterested of course. Dr. Ballingall remarks that, in making out his sketch, he has borne in mind the sentiment, that "he who neglects to record what he imperfectly knows, in the hope that he will one day know it better, and who waits for the fulness of information, will find, too late, that he has forfeited advantages for which no accuracy of knowledge can compensate; and that he is attempting to describe with blunted feelings, that which owes its all to the very imperfection of his knowledge." The remark is but too true, and, if we chose, we could point out some memorable, though perhaps invidious, illustrations in the medical literature of the present day. But we must get us to our task.

It appears that during the season there have been several remarkable examples of tumours within the orbit of the eye.

*Case* 1. In the month of January, 1827, J. M'Intosh was exposed to cold, and, after a certain time, (which is not clearly stated,) noticed a tumour in the right orbit. In July he was admitted into the Royal Infirmary, but would not submit to an operation, and was dismissed. In November he returned, the disease presenting the following appearances: Between the roof of the orbit and the globe of the right eye, there is a soft moveable tumour, depressing the ball of the eye towards the cheek, and pushing outwards the upper eye-lid, which is considerably inflamed, as well as the conjunctiva covering the surface of the tumour. The structure of the eye is apparently sound, and the vision unimpaired, save that the depression of the organ obliges the patient to throw back his head, and elevate his face, in attempting to see objects placed before him. An operation was determined on, and executed by dividing the superior palpebra upwards and outwards from the external canthus of the eye, and having dissected it off from the surface of the swelling, separating the tumour (which was done with much difficulty) from the contiguous parts. A pedicle, by which it sprung from the very bottom of the orbit, was cut across by the probe-pointed scissars, and small portions of it afterwards removed.

For nearly a week the man did well, but at the end of that time a violent erysipelatous inflammation attacked the face and forehead, extending thence over the whole head, and accompanied with delirium, and a very high pulse. These symptoms were somewhat relieved by general and local bleeding, saline purges with antimony, blister to the nucha, and anodyne fomentations: but, on the 22d, he was in the greatest state of prostration, with cold extremities, and muttering delirium. Under the use of stimulants, and another blister to the neck, he again rallied, the cornea ulcerated, and the crystalline lens was discharged through the aperture. The delirium continued with intermissions, he had a ravenous appetite, and on the 28th he expired, his symptoms all along having the typhoid character. No complete dissection was permitted, but a portion of the principal tumour was found still attached to the sheath of the optic nerve, and several small melanotic tubercles imbedded in the fatty matter surrounding the muscles of the eye. Some serous effusion both on the surface and in the ventricles of the brain.

"In the treatment of this case I have no hesitation in saying, that if I had been fully aware of the nature of the disease, and of the deep attachment of the tumor, I should have proceeded at once to extirpate the whole contents of the orbit; but having succeeded in removing the bulk of the tumor with safety to the eyeball, I felt reluctant to change my plan of operation. The inflammation immediately succeeding to the removal of the tumor was, as I have already noticed, much less than was to have been expected from so severe an operation, but when the symptoms of erysipelas supervened, it was obvious that the case became one of a very perplexing and hazardous description.

"The patient's system being surcharged with mercury, we were debarred from the use of mercurial purgatives, which are often so beneficial in erysipelatous inflammation; and this man had been remarked, even when in the hospital in July last, to have something of that sallow cachectic look often attendant upon internal organic disease, and which rendered him, in my estimation, an unfit subject for profuse or extensive evacuations of blood."

*Case* 2. This was under the care of Dr. Hunter, and very similar to the above. The patient was a lad, aged 17, admitted into the Infirmary on the 28th of January, the left eye being pushed close to the roof of the orbit, and protruding from it, the lids separated, and the conjunctiva of the lower everted by a tumour, apparently occupying the whole of the floor of the orbit, of a soft fleshy consistence, but not moveable. There was no head-ache, no pain in the eye, which could be moved in all directions, but a sense of tension in it, and a frequent watering. The sight was a little impaired, but he could read large print. Eight months previously he had received a blow upon the eye, which gave him considerable pain, and in the course of three weeks he noticed swelling in the orbit, which gradually increased. Leeches, blisters, lotions, were employed, and a puncture was twice made under the eye-lid, giving issue to about half an ounce of blood.

The tumour was removed by Dr. Hunter, without injury to the ball of the eye, and was found to present the same general melanoid characters as the former, though it was softer, and in some parts quite liquid, like tar. At the end of a week after the operation the cornea ulcerated, in spite of argent. nit. and vinum opii, and protrusion of the iris took place; erysipelas of the parts around the eye also occurred, but gave way before repeated bleedings ad deliquium, salines with antimony and fomentations.

Dr. Ballingall does not consider these melanoid tumours as malignant, at least when compared with fungus hæmatodes and cancer. The two latter begin in a definite point, and extend their ravages in all directions, invading, at it were, with a poisonous leaven, the tissues in their vicinity, whilst the melanotic matter, on the contrary, is constantly found in a regular cyst. The following appears to be a case of fungus hæmatodes of the eye.

*Case* 3. On the 29th of January, Robert Amos was admitted with a soft elastic tumour, slightly moveable, and about the size of an orange, projecting from the right orbit. Superiorly the finger could be readily insinuated between it and the skin of the eyebrow, but in every other direction it encroached much on the skin of the surrounding parts. The skin of the palpebræ and conjunctiva, which covered the greater part of the tumour, was of a dark colour, and the veins very tortuous, whilst in the centre of the tumour, there was a slightly elevated, sloughy, bleeding fungus, through which the probe passed backwards for two inches and a half, and from which issued dark-coloured fetid discharge. On either temple, and beneath the skin of both lips, were smaller tumours, of similar character, but firmer consistence. Ten years prior to admission the eye was injured by a blow; two years afterwards the sight was gone; and in six years more lancinating pain began, and was succeeded by the swelling, which went on progressively increasing.

The whole of the diseased mass was removed by Dr. B. and the bony margin of the orbit near the external canthus being affected with the disease, was scraped off with a strong scalpel, and the cavity filled with lint. A slight attack of erysipelas followed, but was readily checked; the cavity suppurated, and healthy but pale-coloured granulations sprung up. On examining the eye, the texture of the tumour was found to be so fairly broken down, that it was difficult to say exactly what it was, though the appearances were most like those of fungus hæmatodes. The tumour began in the globe itself, and presented the soft elastic feel of the above disease; but one argument against it was, "the existence of other tumours in the neighbourhood, evidently of a melanotic appearance."

If the disease was really fungus hæmatodes, the chances of ultimate cure must be slight indeed. The cases related by Mr. Abernethy, Mr. Burns, and Mr. Hey, particularly the latter, terminated most unfortunately, even, in many instances, when the disease was seated in the extremities, and that most complete of all extirpations, *amputation* of the member was performed. Dr. Ballingall, however, thinks that he has witnessed two instances of cure, one where the disease was seated in the mamma, the other in the arm. We almost fear, from what we have ourselves seen, that Dr. B. has been deceived.

---

### 93. Clinical Report of La Pitié.*

### *Ligature of Arteries.*

In the commencement of this report there are some general rules laid down for the finding and securing of arteries, but as they are most of them tolerably well known, if not generally acted upon, by surgeons in this country, they scarcely require any notice here. We may mention, however, that M. Lisfranc agrees with the majority of his countrymen in still preferring broad ligatures made of

* M. Avenel. Révue Medicale, Mars, 1821.

vegetable matter, "placed upon a cylinder of diachylon," because they cut through the middle and inner coats of the artery quite as readily as the fine ligature, and have not the disadvantage of destroying the outer coat too soon. M. Lisfranc adapts the breadth of his thread to the calibre of the artery. In this country, surgeons decidedly prefer the fine ligature, because it makes a cleaner incised wound of the inner coats, and certainly has *not* been found, in practice, to destroy the outer a whit too quickly. Whether it is that a *leettle* prejudice, as Mr. Cobbett would call it, still lurks in the minds of our Gallic brethren, or that the arteries of John Bull partake somewhat of the toughness of his native beef, and will stand more tugging and hauling than the soupe-maigre tubes of Monsieur, we leave to keener casuists to determine. But to the cases.

CASE 1. *Fungus Hœmatodes—Ligature of the Carotid—Death.*

Josephine Lenoir, æt 18, of a lymphatico-sanguineous temperament, having previously enjoyed good health, perceived, when twelve years of age, in the right parotid region, a tumour accompanied with cephalalgia, pulsation on that side of the head, and palpitations of the heart. The tumour was thought by the medical men who saw her to be enlargement of the lymphatic glands, but after a-while hæmorrhage began to take place from time to time from the external ear, and the pulsations became more distinct. On the 11th January, 1827, she entered La Pitié, in the following condition. Behind the angle of the lower jaw, stretching beneath the lobule of the ear, was a soft regularly round tumour, about the size of an egg; there was no discolouration, it diminished under pressure, and pulsated strongly and frequently, the pulsations being in time with those of the heart, and diminishing but not ceasing entirely on compression of the common carotid. The disease was thought to be aneurism of the external carotid, and the girl was bled and kept on low diet from January until April. During this interval the heart was examined by several physicians, all of whom pronounced it to be sound, but the carotid pulsated so strongly and extensively, that it was feared the vessel was diseased. A very free hæmorrhage taking place from the ear, a careful examination of the tube was made, and a little soft reddish tubercle found in its interior, which gave rise to the suspicion, that the disease was not aneurism after all, but fungus hæmatodes. M. Lisfranc, distrusting his own judgment, presented the patient to the Royal Academy of Medicine, most of the members of which gave their opinion in favour of aneurism, and all in favour of an operation.

Backed by such authority, M. L. determined on tying the common carotid, if it was sound, and if not, the innominata, as had been previously done by Valentine Mott of New York, and Graëfe of Berlin. Accordingly, on the 10th April, the operation was performed in the presence of several members of the Academy. The steps we need not describe, but the vessel (carotid) seems to have been discovered and tied with facility and dispatch, and the patient bore the whole remarkably well. The tumour, on tying the ligature, diminished to nearly one half, but its pulsation did not entirely cease.

At one, P. M. the pulse got up, with flushed face, and cephalalgia. *V. S. ad* ℥xvj.—*ice to the tumour*. Towards evening the patient began to complain of sore throat, and the ice was discontinued. There was at this time syncope, cephalalgia, pain in the sternum and epigastric region, full pulse, &c. but as night advanced these symptoms wore off, and the girl got some sleep. 11th. Much the same as yesterday afternoon, but a hæmorrhage from the nose occurred this morning, which relieved the head-ache. At present, there is much throbbing in the head and dyspnœa. *Bleeding from the foot to* ℥xvj. The patient was relieved by this bleeding, but it was necessary to repeat it in the evening, and again, to the same amount, on the 12th and 13th. 14th. Heat of skin, pulse full, face injected, oppression at the chest, feeling of great debility. *Bleeding from the foot to* ℥viij. *with slight syncope and relief.* She went on with little alteration until the 17th, when there was such violent cephalalgia and pain in the chest, that it was necessary again to draw blood. In the evening, on dressing the wound, a remarkable pulsation was observed about the ligature. On the morning of the 18th, the patient, after awaking from sleep, complained of thirst, but, in the action of raising her head to drink, a frightful hæmorrhage burst from the wound. The *religieuse* endeavoured to stop it, but in vain, and when the house-surgeon arrived, syncope had taken place. The bleeding ceased, but in little better than an hour the poor girl expired.

*Dissection, 45 hours after death.* The petrous portion of the temporal bone of the right side was triple its natural volume, so as to be on a level with the lesser wings of the os sphenoides. The structure of the bone was soft and friable, its interior spongy and reddish, with open cavities, apparently dilated veins. The extremity of the petrous portion was united with the posterior clynoid processes, and the whole so disorganized, that probes could be passed in all directions, from the interior of the cranium to the neck. The seventh pair of nerves, on entering the internal meatus auditorius, were double their natural size, but after entering the bone, such was the confusion of the diseased parts, that the most careful dissection could trace them no further. The dura mater in the right temporal fossa was thickened and inseparably united to the bone. The petrous and cavernous sinuses of the right side were enormously dilated and filled with an erectile, spongy, cellular substance, but no blood. The arachnoid and pia mater, opposite the middle lobe of the cerebrum, were thickened and adherent to one another. The tumour in the neck occupied the space between the angle of the lower jaw and the mastoid process, and had taken very strong attachments to the inferior surface of the temporal bone and the meatus auditorius. The relations of the tumour to the neighbouring parts were very extensive, and are given with the most praiseworthy minuteness and anatomical precision. However, we shall pass them over, and merely state that the parotid gland was found on its anterior part very much wasted, but unaltered in structure; that the tumour was supplied by the posterior auris artery, which was much enlarged, and by three great branches from the internal carotid which passed through its substance—that a prolongation of the tumour extended *within* the internal jugular vein; and that a great number of new vessels had sprung up, as it were, around it and about it. The structure of the tumour was soft, spongy, of a dirty reddish brown, one part being formed of aneurismatic vessels, the other of an erectile tissue. Within it were bands of fibres forming cells, apparently "only a dilatation of the arteries and veins, losing themselves within it, and constituting the elements of the fungus."

The wound made by the operation was half cicatrized, the other half presented healthy granulations. No abscess, no collection of matter existed in it, and the ligature lay in it perfectly secure. The rupture, in fact, had taken place an inch and a quarter *below* this point, and the intervening space of the artery was perfectly entire, and occupied by a firm coagulum!

This case is certainly remarkable, both on account of the curious nature of the disease, and the still more curious sequel of the operation. It is stiled in the report an instance of fungus hæmatodes, but if we understand the description aright, it looks as much like "aneurism by anastomosis" as fungus hæmatodes. The mode in which the secondary hæmorrhage occurred is the most puzzling of the whole; for we are told, that if we can only get a sufficient plug of coagulum to seal the extremity of the vessel on the separation of the ligature, we are safe, and the danger of hæmorrhage is over. Here there was coagulum enough, and yet the artery gave way, and in a most extraordinary situation too, viz. not where it was bruised and injured by the ligature, but an inch and a quarter lower down! Verily, this is one of those cases which make our fine physiological principles look foolish!

Case 2. *Wound of the Femoral Artery—Spurious Aneurism—Vessel successfully Tied.*

It is now pretty firmly established, that the principle of John Hunter's operation for aneurism is inapplicable to wounds of arteries, and that a single ligature at a distance from the wound, between it and the heart, is bad and dangerous practice. The general rule, and a very excellent rule too, in cases of wounded arteries is—to cut down and tie the vessel above and below the puncture. It has been thought, however, by some surgeons, amongst others Mr. Guthrie, that the ligature of a large artery like the femoral, in cases where no aneurism has previously existed, and the collateral vessels in consequence are not dilated beyond their natural dimensions, is in many instances attended with danger of gangrene, from the sudden check to the circulation in the limb. That there are many cases on record which might be adduced in favour of the opinion, we are ready to allow, but, at the same time, we question if the preponderance of *facts* be not upon the other side. The present case, for one, militates against the doctrine.

M. T. æt. 23, of sanguineous temperament, and violent temper, in a fit of passion, on the 3d of June, 1827, stabbed himself with a kitchen knife, in the lower part of the upper third of the thigh, the blade passing through the limb in the direction of the femoral artery, just above where it is enclosed within the sheath of the triceps. A frightful hæmorrhage took place, and M. T. fell in a second or two bathed in blood. M. M. Devilliers, Salone, and Martinet, arrived in a few minutes, and made a firm compression on the artery in the groin, but this was rendered difficult by constant vomiting on the part of the patient. It was four or five hours before M. Lisfranc could arrive, at which time there was vomiting, subsultus tendinum, attacks of syncope, deadly paleness, the limb double its natural volume, but the integuments undiscoloured. M. L. without delay cut down upon the vessel at the part where it was wounded, and discovered it, apparently with little difficulty. There was no effusion of blood in the sub-cutaneous cellular tissue, nor beneath the fascia lata, but about the sartorius muscle, where it had been cut, extravasation had taken place, with adhesions of the muscle to the neighbouring parts. Beneath the sartorius and the fascia proceeding from it, were found some very dense clots, as large as a hen's egg. They were cleared away and the arterial sheath freely opened, when the artery was found lying quite empty behind the vein which became exceedingly distended during the efforts of the patient. Much difficulty was experienced in discovering the situation of the wound in the vessel, and it was necessary at last to withdraw the compression and allow a gush of blood to guide the surgeon to the spot. This having been indicated, M. Lisfranc first tied the vessel above, taking care that no nervous filament was included in the ligature, and then below, in order to do which he was obliged to divide the sheath of the triceps for about a quarter of an inch. The edges of the wound were brought together by adhesive straps, compresses, and bandage, but there came on so much pain and swelling in the limb that it was necessary to slacken the latter, and indeed remove it entirely from the leg. Next day there was heat of skin, slight delirium, full pulse, &c. but on the 5th, this had quite subsided. The limb always preserved its natural temperature, and on the 10th, pulsation was felt in the popliteal artery. On the 12th, the anterior tibial was felt throbbing, and, on the 14th, the same was observed with the posterior tibial and plantar. On this day the lower ligature came away, and on the 16th, the upper followed. On the 30th, the patient was able to resume his usual occupations, some swelling still remaining about the wound, which, however, was soon dispersed by the application of a few leeches.

Of course it must be an object with all surgeons to avoid including nerves in the ligature with the artery, and it appears that M. Lisfranc is in the habit of guarding against this mistake, by slightly rotating the vessel on its axis with the point of his finger, so as to ascertain that it is completely free, before he ties the thread. M. Lisfranc objects to the term, "false primitive aneurism," as applied to these cases, because it often happens that the greatest effusion of blood into the neighbouring parts is not immediately upon the reception of the wound, but at a later period. M. L. has seen simple gun-shot wounds, uncomplicated with broken bone, followed by false aneurism *fifteen* or *twenty* days after the injury, but surely the rupture of the vessel in this case must be from *ulceration*. We think, ourselves, that the designation of "diffused aneurism," which is generally applied in this country is the best, as it involves no theory, but merely states an obvious fact.

CASE 3. *Sarcoma of the Lower Jaw—Amputation of half the Bone—Cure.* Monsieur Lisfranc is a bold surgeon and cool operator, as this case will shew. By the bye, we fear that the remarks which have been lately made on the above gentleman, M. Roux, and the officers of the French hospitals generally, in the Medical Gazette, will do no good. English practitioners and English students have always been received with politeness and liberality at the Parisian hospitals, and the publication of strictures, many of which are any thing but good natured, upon the practice of the professors, is making but a sorry recompense for favours received. It is like betraying your host after eating his bread and salt;

> "the salt, that sacred pledge
> Which, once partaken, blunts the sabre's edge,
> Makes even *contending* tribes in peace unite,
> And hated hosts seem *brethren* to the sight!"

P. F. Vaneisam, æt. 47, of sanguineous temperament, and good constitution, entered the Hospice de Perfectionnement, Nov. 9th, 1827, with a sarcomatous tumour, about the size of two large eggs, extending from the left ramus of the inferior maxillary bone, to the symphysis. The gums were affected with a cancerous ulcer, the discharge from which was sanious, and extremely fetid; the lymphatic glands beneath the jaw on the affected side, were considerably enlarged, the integuments entire. Seven months previous to his admission, the patient first noticed a small tumour connected with the bone, about the size of a common pea; this gradually increased, attended with continued lancinating pains, and obstructing both articulation and mastication. M. Lisfranc, prior to the performance of any operation, made a trial of antiphlogistic measures, which dispersed the enlargement of the lymphatic glands, but had no effect whatever either on the size of the tumour or the lancinating pains experienced in it. Accordingly, on the 26th of November, the disease was removed by the knife in the following manner.

An incision was begun in the centre of the lower lip, and carried perpendicularly downwards to the lower margin of inferior maxillary bone, quite through the soft parts covering it. In consequence of the volume of the tumour, it was found necessary to prolong this incision an inch lower down, from which point the knife was continued transversely, as far as within a quarter of an inch below and before the angle of the jaw. The fingers of an assistant had been previously placed on the "origin" of the carotids to mark their situation, and from the lower border of the chin to the angle of the jaw, the incision was merely carried through the skin and cellular membrane. The dissection of the soft parts from the surface of the tumour was extremely painful, but the flap having been formed, two teeth were extracted, the bone was divided across the ramus by a fine saw with extraordinary facility, and then the symphysis by a common saw; the operator seized the upper part of the tumour with his three middle fingers, the lower with his thumb, and then, by means of slight force, was able to draw down the jaw, and separate it from a great quantity of the tissues attached to it behind. The tumour was found to extend deeply beneath the tongue, and required a very cautious dissection to remove it. The ranine arteries were not wounded, indeed no vessel of any consequence, so that *not a single ligature nor cautery was applied!*

Three hours after the patient had been placed in bed, M. Lisfranc drew together the lips of the perpendicular incision by the interrupted suture, but did not attempt to unite the transverse portion of the wound, in order that liquids might flow readily. On the 31st, the perpendicular portion had united, and the lower was freely suppurating. On the 15th December, the patient could speak with facility, and was allowed solid food. Saliva continued to flow through the lower wound, and compression was made upon its edges, but it caused too much pain and was discontinued. M. Lisfranc then placed in the mouth a small sponge to absorb the liquids, and in the course of a few days the wound had entirely cicatrized. The patient was kept in the hospital a month after this, and then departed for the country, the distortion of the jaw being very slight, indeed the deformity scarcely apparent on wearing a cravat. The patient could articulate distinctly.

In the remarks upon the case, M. Lisfranc details the appearances observed in patients who have died some time after amputation of greater or lesser quantities of the lower jaw. If the portion removed be small, and taken from the median line, the portions are little removed from each other and unite like a common fracture. 2ndo. When the ends of bone remain at a certain distance, asunder, a very solid fibrous substance like that which unites a badly set patella, is found to intervene between them and prove a considerable check upon their motion. 3tio. In cases when the union between the ends of bone is firm, nothing particular happens to the temporo-maxillary articulation, but when, from the action of the muscles, the inferior extremity of the bone is carried inwards, luxation, partial or complete, of the condyle of the jaw from the articulating cavity is the consequence.

This concludes M. Lisfranc's Clinical Report, and a very good one we must confess it is. We think it is impossible to peruse it without being struck with the boldness and dexterity of the operator, and again we take an opportunity of deprecating the mode of commenting on this and other French surgeons, adopted by the Parisian REPORTER in the Medical Gazette. Its effect, if it has any, must be to engender ill-blood, and perhaps deprive English students of advantages in Parisian hospitals which they are at present in possession of. As we before observed, we are sure that it is not the wish of our contemporary to do any thing that is not liberal and gentlemanly, but, at the same time, we must say that the spirit of his REPORTER smacks rather too much of the leaven of a certain Journal that need not be named,

---

94. SECONDARY HÆMORRHAGE.

*Case* 1. A man of rather spare habit, was admitted, April 10th, into St. Thomas's Hospital, under the care of Mr. Travers, with compound fracture of the olecranon, and dislocation forwards of the radius and ulna in consequence of a blow from the handle of a crane. So much violence had been already received, that it was not judged prudent to inflict any more *secundum artem*, by attempts at reduction; the limb was accordingly laid out in Mr. Amesbury's apparatus, and leeches applied, in spite of which, inflammation and suppuration in the joint very rapidly took place. Under these circumstances, amputation of the arm was performed on the 26th, four arteries requiring ligatures. On the 27th, pain and heat of the stump came on, and on the 28th, the bandage being found, on account of the swelling, to have become very tight, was removed. Next day, the other dressings were removed also, when the stump looked foul and sloughy, and the discharge was copious and unhealthy. *Poultices—nutritious diet.*

Within eleven days after the operation, all the ligatures except that on the brachial artery had come away, but on the 6th of May, there was suddenly a gush of arterial blood from the stump. Pressure was applied, and when Mr. T. arrived, the hæmorrhage had ceased, but the patient was much enfeebled, and the pulse decidedly hæmorrhagic. Mr. Travers, with great difficulty, in consequence of the ragged, sloughy condition of the stump, removed the ligature from the brachial artery and re-secured it, but next morning the bleeding re-appeared. Mr. South was called, and finding it to proceed from the profunda artery (which?) attempted to tie the vessel, but from its being implicated in the surrounding mass of disease, was obliged to include some of the muscle, &c. in the ligature. After a few minutes the same vessel again burst out bleeding, and was again secured, but rather higher up. It was of no avail, for the patient sank next day.

On opening the chest serous effusion, with flocculi of lymph was found in the left cavity of the pleuræ, both of which were thickened, opaque, and coated with a layer of coagulable lymph. No examination of the vessels of the stump was made. *Med. Gaz.*

We cannot say that we much admire the practice pursued in this case, or the dissection of it afterwards. As far as we can perceive, and really we make no pretensions to seeing farther than our neighbours, the hæmorrhage was owing to the sloughing which came upon the stump, and extremities of the vessels which lay in it. If such *was* the case, what hope could there be in pulling one ligature *off* a diseased vessel, only to put another ligature *on?* Would it not have been preferable to have tied the brachial artery *above*, instead of diving with needle after needle, and irritating parts already irritable enough? We mean, of course, no reflexions on the surgeons, for whose professional attainments we have the highest possible respect, but, as we are told that Homer *sometimes*

nods, even so, we suppose, may Mr. Travers. In the Number of the Gazette succeeding that in which the above appears, Mr. South suggests transfusion, and the reporter asks, what would have been the effect of it? We think we could very safely answer—no good, if it had any effect at all! Mr. South also hints at the actual cautery, and relates a case which happened to Mr. Cline, jun. where, after amputation of the thigh, the stump became unhealthy, hæmorrhage took place, and was arrested by the actual cautery. We cannot say that we entirely agree with the proposal, indeed if we might be allowed a bad joke, we should say that Mr. South, has got out of his *latitude*.

Case 2. *Removal of Carcinomatous Glands from the Groin—Secondary Hæmorrhage—Ligature of the External Iliac.*

James Thompson, æt. 39, a chimney-sweeper, was admitted into the Royal Infirmary, Sept. 10th, and soon afterwards some carcinomatous glands situated close upon the great femoral vessels, were removed by Dr. Cullen. The wound went on granulating kindly for the first fortnight, and was much reduced in extent, when suddenly it assumed a sloughy or phagedænic appearance, and became excavated to a considerable depth. In this state, it continued stationary for several weeks, and then again took on the ulcerative process, with severe lancinating pains, œdema of the limb, and much constitutional disturbance. Laxatives and opiates with poultices, and the arsenical solution locally, were the means employed, but about the middle of December, some slight discharges of arterial blood were observed to issue from the wound.

On the morning of the 18th, there was an alarming bleeding, which recurred again in the course of the forenoon, but was readily suppressed by the application of dry lint with pressure. In the afternoon, there was hæmorrhage to the extent of several ounces; pressure by the hands of an assistant was directed to be kept up, and, at half-past seven, P. M. it was determined in consultation to tie the external iliac. This was done by making an incision, straight in consequence of the encroachment of the ulcer on the lower margin of the external oblique, from near the abdominal ring to the anterior superior spine of the ilium. After dividing some thickened and indurated cellular membrane, the tendon of the external oblique was exposed, and divided with the internal oblique and transversalis, "when the artery was readily found pulsating at the bottom of the wound, with some enlarged lymphatic glands lying over and contiguous to it." In separating the glands, a large vessel was ruptured and bled smartly, but the iliac trunk having been seized, above the bleeding point, between the fore-finger and thumb of the left hand, the aneurism-needle was passed under it with the right, the knot tied, one end of the ligature cut off, and the wound dressed with adhesive strap. As nothing whatever is said of the peritoneum in describing the steps of the operation, we suppose it must have got out of the way, which was certainly very obliging on the part of that membrane. The ruptured vessel must have been either the epigastric or circumflexa ilii.

Next morning there were symptoms of slight peritoneal inflammation, which was treated by leeches to the abdomen, mild laxatives and fomentations. The pulse, for the ten days subsequent to the operation, ranged from 90 to 110, and on the eleventh day the ligature separated, at which time great part of the wound had united, the œdema of the affected limb was considerably diminished, but there was still a great degree of numbness, and no pulsation in any part of it. On the 30th January the wound had nearly cicatrized, and the patient was getting up, when hæmorrhage took place, and recurred in the afternoon, but was checked by a piece of sponge. On the morning of the 31st, two slight bleedings, hæmorrhage to the extent of two pounds, "apparently from a branch of the obturator, the ulceration having latterly extended down towards the thyroid foramen." The man had nausea, fluttering pulse, &c. and the flow of blood was stopped by sponge and pressure, which were continued until the 10th February, it being determined in consultation that no further operation should be attempted. The bleeding has not returned, but the ulcer is spreading, and the prognosis none of the most favourable.

If any one case is more perplexing and distressing to a surgeon than another, it is that in which secondary hæmorrhage occurs to mar his operations, destroy both the *physique* and the *morale* of his unfortunate patient, and undo in a few hours what Nature and he have been days or even weeks in getting done. We have chapters, and essays, and disquisitions, and pretty little theories by the score, upon the causes and nature of secondary hæmorrhage; but there is something rotten in the state of Denmark after all, and, in very many cases, the why and wherefore of the bleeding are any thing but clear. When it occurs a few hours after an amputation or other operation we can understand it, and so we can, or think we can, when it follows hard upon the separation of a ligature from a great arterial trunk. But when, as sometimes happens, the stump is almost healed, the ligature has come away for a fortnight or three weeks, and suddenly a gush of blood tears up, we might almost say, the cicatrix of the wound, when the hæmorrhage takes place in this manner, it would puzzle even the most "bookish theoric" to explain the quo and quomodo. The case which occurred some time ago at St. George's Hospital, and which we related at the time, furnished a good illustration of the difficulties which environ this interesting subject. In that case, a ligature was applied on the femoral artery for popliteal aneurism, which separated without the least accident at the termination of a fortnight. *Fourteen* days after this, hæmorrhage suddenly occurred, and the artery was tied higher up. This second ligature remained upon the vessel for three weeks, and all was doing well, when suddenly, *thirteen* days after its separation, hæmorrhage again took place, and ultimately destroyed the patient. There was no sloughing of the wound, no ulceration, then why should the artery give way, in both instances, almost on the very same day?

---

95. MARCH OF INTELLECT—RETROSPECTION.

We do not see why the "march of intellect," like the march of an army, should not be sometimes retrogressive as well as progressive. The history of the human species indeed teaches us that intellect has been marching and countermarching from the earliest periods down to the present moment. Nor is a retrograde movement always unwise or dishonourable. Moreau's *retreat* through the Black Forest did him more credit than all his victories. Even Napoleon's return from Russia was, in our humble opinion, a wiser step, and the result of *cooler* reflection, than his proud and pompous march into that distant country. But however this may be, there can be no harm in casting a retrospective glance at the "march of intellect," in our profession, in order to see what useful things we have picked up or lost on the journey—what errors we have discarded —or what we have still allowed to remain undisturbed.

Walking up the Hampstead-road, the other evening, to inhale the fresh atmosphere of that place, and leave, for a moment, the dust, smoak, din, and distraction of the metropolis behind, we stopped to take breath opposite an humble stall, which, nevertheless, presented three different species of refection—old books, oysters, and soda water. The *first* was the only kind of food for which we had an appetite, and it appeared withall the cheapest—a matter of some consideration in these times. A small volume, minus one side of the cover, and marked "THREE-PENCE," attracted our attention. Its title ran thus:—"The STATUTES of the COLLEGE of PHYSICIANS of London, worthy to be perused by all men; but more especially by Physicians, Lawyers, Apothecaries, and Surgeons—and all such that either do, or shall study, Profess, or Practise PHYSICK. Anno Domini, 1693." Ill as we could afford the expence, we purchased the book; and, having derived considerable amusement, if not instruction, from the volume, we have tried to turn the purchase-money to some little account, and to see whether these same STATUTES might not be converted into a more saleable *article*, by a little brushing and scouring.

It somewhat surprised us, who have great veneration for the "wisdom of our ancestors," to learn that, so far back as 1693, there were corruptions in Colleges—and patriotic reformers to expose them. The editor dedicates his work to PARLIA-

MENT, "in whose determination the people of England, calmly, contentedly, and conscientiously acquiesce."

"Tho the corruptions and perversions of ill men have frequently subverted your most Generous Designs for the Publick Good, to their own base and private Interests; among the number of which, it were heartily to be wish'd that the most Noble and Necessary Art of *Physick* were not prostituted and perverted by the avarice, pride, and oppression of a few, who not content privately to enslave and abuse men, by exacting, &c. &c."

We almost fancied ourselves once more in the Freemason's Tavern, listening to the patriotic effusions of a modern editor and reformer; but we soon found that the periods were widely distant, though sentiments and circumstances were nearly the same.

It appears that the Editor of the Statutes was an ill-used man in his time. He was a practitioner in PHARMACY; but naturally wishing to rise in his profession, he entered himself at Oxford or Cambridge, and, after a time, presented himself before the College of Physicians, and was three times examined by the President and Censors, with satisfaction to all parties. This preliminary over, he "visited all the Fellows, in order to be admitted Licentiate," and was graciously received, but requested by the said Fellows "to suspend his admission of Licentiate till such time as he had quitted his employment in pharmacy, and completed his degree (being then Bachelor in Physic) of Doctor in the University." He was assured that *then* he would be received, without any further examination, let, or hindrance. The good easy bachelor acquiesced, and completed his degree in the English University; but, to his astonishment, found that a new bye-law had been enacted, in the interim, by which he was cut off from the licence!

Now we adduce this hard case, in order to demonstrate the "march of intellect," as well as the progress of more liberal sentiments. Such an occurrence could not, would not, take place in these enlightened days. The licence—nay, the fellowship would not be denied, under such circumstances, in the present times.

But we must now glance at the Statutes, including the bye-laws, of the College, as existing in 1693, and see whether the "march of intellect" has been steadily progressive, or occasionally retrogressive, since that period. The first extract which we will make contains information that is of some importance.

"Let all the Statutes be divided into three parts or sections, the first part comprehends all those things which belong to the Electors, to the President, and Pro-President, to the Consiliarii or Assistants, to the Censors or Governours, to the Comitia or Meetings, and other things, till you come to the Ninth Chapter, which treateth of the Office of the Treasurer.

"The second part comprehends those Statutes which belong to the Treasurer and Register, to the number of Fellows and Candidates, to their Examinations, Elections, and Admissions, to the Order of the Permissives, *and their Office*, and the other things, even to the Two and twentieth Chapter, which treateth of the Penal or Moral Statutes.

"The third part shall contain the Penal or Moral Statutes, and according to this threefold partition of Statutes, they may be read thorow in all greater Meetings, except on the day of the Meetings of St. Michael, at which time the Election of Officers are appointed.

"Let the Fellows, Candidates, and *Permissives*, be present at the Reading of the Statutes, as many as can be there."*

Now, from this document it is evident that, a century and a half ago, the Licentiates were summoned, in common with the Fellows, to hear the President read the laws common to the whole College as well as applying to themselves. Here then there was some connecting link left between the Licentiates and the College —the wall of brass had not been raised between them and the Fellows—and they were monthly made acquainted with all laws touching themselves or their brethren. Considering that the Licentiates, at that time, were characterised as foreigners—as men not graduated at Universities in this kingdom—as men "not eminently learned," &c. we do think that the statute which summoned them to the College, in common with the Fellows, to hear the laws of the insti-

* "Lectioni Statutorum intersint Socii, Candidati et *Permissi*, quot quot adesse Poterunt," p. 50.

tution read, was much more liberal and politic, than that which afterwards excluded them from all shew of connexion with the said College. When the Parliament itself is voting for the removal of disabilities, grounded on ancient prejudice, would it not become the enlightened President and Fellows of the College to open the doors of that edifice, and admit, on some kind of terms, a large class of physicians, against whom there is not the shadow of plea, as to want of learning, talents, or moral worth ? There is a returning disposition to peace and amity among all ranks of the profession, the consequence of re-action against a vile and demoniacal furor (ira furor brevis est) which made a temporary impression on their minds—and this is the time which should be seized by the College, to unite their Licentiate brethren with themselves, by a league befitting the spirit of the times, and the progress of liberal sentiments.

The following Statute will excite a smile in these days.

"Let none teach the People Medicines, or tell their Names to them (especially if they are Vehement Medicines, as Purgers, Opiats, or Sleeping Medicines, or which cause Abortion, Vomits, or any other of greater moment or danger) lest by the abuse of them the People be injured ; upon penalty of forty Shillings as often as they shall offend.

"He that bargains with Apothecaries for any part of the Price arising from the Medicines to be prescribed, shall be fined forty Shillings, as often as he shall so offend.

"Let no Fellow Candidate or Permissive, make use of those Apothecaries, who either themselves Practise Physick, or frequently serve those Physicians, who are not Examined and Approved, according to the Statutes of this Kingdom, under penalty of Ten Shillings, to be paid to the Colledge."

If the above excite a smile, the following will certainly call forth a loud laugh.

"Because Apothecaries and Chirurgeons, but especially the *Chirurgeons*, often bring the Urine of Sick People to Physicians, and desire that from the Inspection of the Urine they would prescribe something for their Sick ; and under this pretence of Consultation, manage the whole Course of Cure at their pleasure, *whatever profit or gain there is ;* appropriating all to themselves, but bringing to the Physicians nothing besides that *slender and hungry reward of inspecting the Urine.*"

"Therefore, We admonish all Physicians, that for the future, they carry themselves much more wary in this matter, than heretofore was wont to be done of many; and for this Reason we forbid all Persons Practising Physick, that they do not prescribe any Medicine to those *Idiots and Silly Women who carry the Pispots of Sick Persons about*, unless they have first known the Sick Person."

The "march of intellect" is no where more conspicuous than in the contrast exhibited between the following statute and the present practice.

"Because it is found by Experience that the Apothecaries from the Prescripts of Physicians, *attain some pretence or shadow of false Knowledge* (everywhere boasting themselves to Sick Persons) which they abuse, not without the hazard of others, to their own profit; We Determine and Ordain, that no Colleague for the future, either Fellow, Candidate, or Licentiate, add Directions as they call them, to their Prescripts or Physical Scrowls (save only in Hospitals) but he shall leave them with the Sick, or at least take care that they be first signed and carried to his house ; in the mean time let him command, that the Medicines prescribed in the Scrowls be only signed with some agreeable titles or apt notes for their distinction, *to the intent that as much as in us lies, nothing at all be smelt out by this sort of Medicasters, with what design, intention, or for what uses the Remedies are prescribed.*"

A perusal of the above and many other statutes abundantly demonstrates how much things are now altered from what they were a century or two ago—and we entreat the president and fellows to reflect on the impolicy, not to say injustice, of still keeping up the rigid exclusion of the licentiates from all participation in the rights, privileges, honours, or offices of the College. It is with them as with the Catholics. A more liberal policy must one day be adopted—and there never was—there never will be, a more favourable moment than the present, for uniting two great classes into one harmonious body—with detriment to neither, and with certain advantage to both.
